# Statistical assessment of discriminative features for protein-coding and non coding cross-species conserved sequence elements

**DOI:** 10.1186/1471-2105-10-S6-S2

**Published:** 2009-06-16

**Authors:** Teresa M Creanza, David S Horner, Annarita D'Addabbo, Rosalia Maglietta, Flavio Mignone, Nicola Ancona, Graziano Pesole

**Affiliations:** 1 Istituto di Studi sui Sistemi Intelligenti per l'Automazione, CNR, Via Amendola 122/D-I, Bari, Italy; 2 Dipartimento di Scienze Biomolecolari e Biotecnologie, Università di Milano, Via Celoria 26, Milano, Italy; 3 Dipartimento di Chimica Strutturale e Stereochimica Inorganica, Università di Milano, Via Celoria 26, Milano, Italy; 4 Dipartimento di Biochimica e Biologia Molecolare, Università di Bari, Via E. Orabona 4, Bari, Italy; 5 Istituto Tecnologie Biomediche, CNR, via Amendola 122/D, Bari, Italy

## Abstract

**Background:**

The identification of protein coding elements in sets of mammalian conserved elements is one of the major challenges in the current molecular biology research. Many features have been proposed for automatically distinguishing coding and non coding conserved sequences, making so necessary a systematic statistical assessment of their differences. A comprehensive study should be composed of an association study, i.e. a comparison of the distributions of the features in the two classes, and a prediction study in which the prediction accuracies of classifiers trained on single and groups of features are analyzed, conditionally to the compared species and to the sequence lengths.

**Results:**

In this paper we compared distributions of a set of comparative and non comparative features and evaluated the prediction accuracy of classifiers trained for discriminating sequence elements conserved among human, mouse and rat species. The association study showed that the analyzed features are statistically different in the two classes. In order to study the influence of the sequence lengths on the feature performances, a predictive study was performed on different data sets composed of coding and non coding alignments in equal number and equally long with an ascending average length. We found that the most discriminant feature was a comparative measure indicating the proportion of synonymous nucleotide substitutions per synonymous sites. Moreover, linear discriminant classifiers trained by using comparative features in general outperformed classifiers based on intrinsic ones. Finally, the prediction accuracy of classifiers trained on comparative features increased significantly by adding intrinsic features to the set of input variables, independently on sequence length (Kolmogorov-Smirnov P-value ≤ 0.05).

**Conclusion:**

We observed distinct and consistent patterns for individual and combined use of comparative and intrinsic classifiers, both with respect to different lengths of sequences/alignments and with respect to error rates in the classification of coding and non-coding elements. In particular, we noted that comparative features tend to be more accurate in the classification of coding sequences – this is likely related to the fact that such features capture deviations from strictly neutral evolution expected as a consequence of the characteristics of the genetic code.

## Background

The annotation of whole genomes through the identification of protein coding and regulatory regions is one of the major challenges in the current research in molecular biology. In comparative genomics, the key idea is that sequences, which are highly conserved during evolution, likely correspond to either protein coding exons or regulatory motifs [1]. In our study, we focused on homologous conserved sequences among three mammalian species: human, mouse and rat. Subsequent to their divergence, these genomes have independently accumulated changes including insertions, deletions and substitutions of nucleotide bases. Comparative genomic studies have found that about 1 billion of the 3 billion bases in each of the genomes of rats, mice and humans align with each other. These aligned bases are thought to be an "ancestral core" that has been retained in the three species. This core composed of 1 billion bases encodes nearly all the genes and their regulatory signals, accounting for the similarities among mammals. However, only a portion of this core constituting 5–6% of the whole genome appears to be under selective constraint in rodents and primates, while the remainder appears to be evolving neutrally [2-5]. Most of coding exons and regulatory elements are included in this highly conserved genome core. In consideration that we still do not know the complete gene inventory of human and other eukaryotic genomes, we are in principle unable to unequivocally assess if a conserved sequence element (CSE) is coding or not basing the decision only on the comparison with the current gene annotation. Indeed, a CSE may well overlap with a still unknown coding exon. The vast majority of coding sequence annotations are derived at least in part from sequence similarity to previously annotated sequences – propagation of "conserved hypothetical protein" annotations thus risks erroneous protein gene predictions. Therefore, we are interested in discriminating between coding and non-coding sequences in this highly conserved genome core, independently of the currently available gene annotation.

We stress the ongoing importance of sequence and evolutionary-dynamic-based discriminators in the prediction of coding genes and the identification of regulatory elements. Different discriminative approaches have been proposed which are based on various measures of the *coding potential*, i. e. measures of the likelihood that sequences with a particular nucleotide substitution pattern or with a certain bases composition are coding sequences. These metrics aim to capture different signals that distinguish coding and non coding conserved sequences and may use comparative or non-comparative features. The former are based on cross-genomic comparisons, whereas the latter are computed by analyzing single-species sequences. The most common comparative features are based on evolutionary signals which aim to quantify 1) the tendency of nucleotide insertions and deletions to preserve the codon reading frame [6,7] and 2) mutational biases towards synonymous codon substitutions and conservative amino acid changes [8,9] unique to homologous coding regions. Concerning the most common non-comparative features, some metrics are based on base compositional bias [10], on asymmetry of the base composition in the three codon positions [11,12] and other quantify the three-base periodicity in genetic code [13,14]. Although different studies exist in literature about the evaluation and comparison of discriminative metrics based on single-species sequences [15,16], a complete study concerning both comparative and non-comparative features is still missing. In the field of comparative genomics, many features have been proposed [17] but a critical study concerning their combination and influence on learning machines in predicting coding and regulatory motifs lacks. In [18,19], the authors combined two measures in a single one without addressing the problem of adding new features and measuring their relevance on the final classifier. In [20], the authors trained Support Vector Machine classifiers on a set of 180 features without focusing on the redundancy of subsets of the features adopted. Moreover, these studies did not address a critical study concerning the influence of the sequence length on the classification performance.

As far as we know an unbiased statistical assessment of the capacity of single as well as groups of features of classifying sequences between coding and non coding CSEs lacks. Many experimental conditions and procedures for estimating the generalization error [21] strongly influence the evaluation of the predictive ability of features and so must be carefully taken into account. In particular, an objective comparison and evaluation of the competing metrics requires an accurate choice of data sets in terms of balance in the sizes and in similarity of sequence lengths in the two classes.

In this paper we have provided a systematic and unbiased statistical assessment of comparative and non comparative features for discriminating coding exons from regulatory motifs. In particular, we assessed the differences of distributions by using Wilcoxon-Mann-Whitney non parametric tests [22] and we estimated the classification ability of single as well as groups of features by using multiple cross validation strategy, which provides an unbiased estimate of the generalization error of learning machines [23,24]. The statistical significance and power of the estimated prediction accuracy of Fisher's linear classifiers were estimated by using non parametric permutation tests [25,26]. In particular, in our study we evaluated the influence of the sequence length on the prediction accuracy of classifiers trained on balanced data sets. Moreover, by using Kolmogorov-Smirnov non parametric test [22] we investigated if adding non comparative features to the comparative ones could improve in a statistically significant way the performances of the classifier. We considered features already reported in literature as well as novel features that attempt to capture extra signals of coding potential.

## Methods

### Data set description

Homologous genes have been extracted from Homologene database selecting only those genes with an annotated reference mRNA (NM_ID) and protein (NP_ID) in all the three organisms considered: human, rat and mouse. Reference mRNA sequences of these genes were mapped on corresponding genomes using BLAT (the BLAST-Like Alignment Tool [27]), then we identified genomic regions corresponding to coding sequence by parsing the BLAT output and the relevant mRNA Genbank entry.

To generate the three-species coding CSE set we run the BLAT search on genomic sequences masked in all non-coding sequences. Conversely, to generate the non-coding set, all annotated coding regions and repetitive elements were masked. In this way the coding set included CSEs corresponding to coding exons, whereas the non-coding set included 5' and 3'UTRs, introns or other intergenic unique regions.

The hortologous genomic sequences of the coding and noncoding set were pairwise aligned by using the BLAST algorithm [28] to generate coding and noncoding conserved sequences, respectively. Conserved core regions shared by all three organisms were extracted and multi-aligned by ClustalW program (with default parameters) generating our coding and noncoding multi conserved data sets consisting of 32318 coding and 5438 non coding alignments.

The length distributions of the sequences in the two data sets (coding and non-coding sequences) are very similar: their lower quartiles, medians and upper quartiles are respectively 83 nt, 114 nt, 154 nt in the coding data set and 74 nt,119 nt,198 nt in the non coding data set.

### Discriminative features

In this section, we provide a detailed description of the measures we chose to reveal the differences between the two classes.

#### Comparative features

The most common comparative features are based on evolutionary signals as mutational biases towards some codon substitutions. The evolution of highly conserved sequences, both coding and non-coding, is under the control of negative selection. However, their evolutionary dynamics is expected to be quite different for these two classes of sequences. Due to the nature of the genetic code the majority of base substitutions in coding regions tend to be synonymous [29], thus mostly affecting the third codon position, with non-synonymous changes favoring interconversions between amino-acids with similar chemical-physical properties. On the other hand non-coding conserved sequences follow a completely different evolutionary dynamics as negative selection, in this case, acts to preserve the binding of regulatory proteins (e.g. transcription factor binding sites) or regulatory RNAs (e.g. miRNAs) [8,9]. To quantify these differences, we evaluated the following metrics.

#### Rate ratio

Following the Nei-Gojobori approach [29], we computed the number of codon pairs which differ only for the nucleotide at the i-th position, i. e. the number of single base changes:



and the number of codon pairs which differ for two or three nucleotide differences one of which is at the i-th position, i. e. the number of multiple base changes:



*S*_*i *_among the  substitutions are synonymous. Assuming that the proportion of substitutions in multiple bases which are synonymous is equal to the estimated proportion of synonymous substitutions among all substitutions in single bases , we computed the number of synonymous substitutions:

(1)

and the number of nonsynonymous substitutions

(2)

To compare the two numbers *S*_*d *_and *N*_*d*_, we must differently weight them because the number of potential synonymous sites is much smaller than the number of nonsynonymous sites.

To this end, we computed the numbers of synonymous and nonsynonymous substitutions for each codon position, i. e. for the i-th codon position we evaluated the proportion *s*_*i *_of possible substitutions in the i-th codon position which are synonymous and the proportion *n*_*i *_of possible substitutions in the i-th codon position which are nonsynonymous (*n*_*i *_= 1-*s*_*i*_). We normalized *S*_*d *_and *N*_*d *_by using the average of *s*_*i *_and the average of *n*_*i *_over all pairs of aligned triplets, denoted S and N respectively, obtaining the following quantities:

(3)

known as p-distances [30]. In the study of the evolutionary divergence, the above computed p-distances are corrected to account for multiple substitutions at the same site by the Jukes-Cantor correction and become

(4)

known respectively as synonymous substitution rate and nonsynonymous substitution rate [31]. The  rate ratio is used as measure of the relative importance of evolutionary forces that have shaped a particular protein. A rate ratio significantly greater than one strongly suggests that positive selection has acted on the protein: the nonsynonymous substitutions are "relatively" more frequent. The values *d*_*s *_and *d*_*n *_are defined only if *p*_*s*_and *p*_*n *_are smaller than . So we preferred to neglect the Jukes-Cantor correction and to use the p-distances. Moreover, the ratio  or equivalently  becomes infinite when its denominator is zero. In order to avoid this problem, we defined as measure of selective pressure the following substitution rate ratio:

(5)

where *p*_*s *_is the estimated proportion of synonymous nucleotide substitutions per synonymous sites and *p*_*n *_denotes the estimated proportion of nonsynonymous nucleotide substitutions per nonsynonymous sites as above defined. Our measure is the proportion of synonymous nucleotide substitutions per synonymous sites normalized by using the sum of both proportions of the nucleotide substitutions. A SRR ratio of 0.5 suggests that these genes have evolved without constraints, a value of SRR greater than 0.5 (*p*_*s*_> *p*_*n*_) suggests that nucleotide substitutions that don't change the encoded amino acid (negative selection) are the most frequent substitutions. On the contrary, a SRR smaller than 0.5 (*p*_*s *_> *p*_*n*_) indicates that the most frequent substitutions are those which change the encoded amino acid (positive selection). Note that, in the computation of SRR in 5, we consider only the aligned triplets without gaps. MRna sequences which differ at the most for gap triplets have SRR equal to infinite. These sequences are so similar that we can't establish if the selective pressure is positive, neutral or negative, so we excluded these sequences from our study. The measure SRR clearly depends on the reading frame and for the protein coding sequences there is only one correct reading frame. We expect the SRR gets the maximum value in correspondence of the correct reading frame. To this end, we chose as coding potential score the maximum value of SRR out of six possible reading frames. On the contrary, for the non coding sequences all the six reading frames should have similar SRR values and so a smaller dispersion on the different frames around the maximum value. For this reason, we evaluated this dispersion as follows:

(6)

(7)

#### Blosum score

In order to quantify amino acid similarity between the two aligned sequences, we averaged over the Blosum scores of consecutive triplet pairs for each reading frame *BL*80(*i*) [32]. We used a modified version of the BLOSUM80 by assigning a null Blosum score to the couples of two stop codons and to the couples of codons of which one has three gaps codons and the other one hasn't any gap or has three gaps and a Blosum score of -9 to couples of codons with almost one codon with one or two gaps [9]. As metrics of the coding potential, we selected the maximum value on the different frames and its coefficient of variation around the maximum:

(8)

(9)

Note that, to ensure that the coefficient of variation wasn't infinite, we added 9 to the *BL*80_*M *_value at the denominator.

#### Reading frame conservation

**Figure 1:**
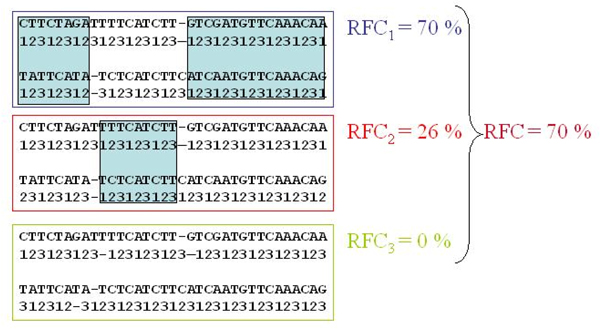
**The reading frame conservation**. This figure shows the reading frame conservation test by M. Kellis et al. (2004).

In coding exons of conserved regions, alignment gaps don't shift the reading frame (id est gap lengths are multiple of three bases) or are arranged to let the recovery of the frame. We evaluated the percentage of nucleotides that are in the same frame for each pair of the sequences in the alignment and for each possible offset:

(10)

(see Figure 1). In detail, we labeled the nucleotides of the first sequence (skipping the gaps) by their codon position beginning with the first one, and labeled the nucleotides of the second sequence beginning once with the first, once with the second and once with third codon position. Then we counted the percentage of nucleotides equally labeled in each pairwise comparison *RFC*_*i *_and selected the maximum value *RFC *[6]. We expected that this value was greater in the coding sequences data set than in the non coding one.

#### Nucleotide Percent Identity

Finally, we considered the percentage of bases which are conserved across each pair of sequences denoted by Nucleotide Percent Identity (NPI).

### Intrinsic features

In the following, we describe the coding potentials which were derived from single species sequences.

#### C+G content

It's well known that the concentration of genes is correlated with a highest C+G density [10]. For this reason, we counted the C+G content in each sequence in analysis.

#### Percentage of stop codon

Moreover, we expected a smallest percentage of stop codons in the correct reading frame of a protein coding sequence (just one codon stops the translation) than in a non coding sequence, where the triplets have no meaning for the amino acid translation. So we counted the percentage of stop codons (TAA, TGA, TAG) in each reading frame %*Stop*(*i*) and selected the smallest such percentage out of six possible reading frames and its dispersion around the minimum:

(11)

(12)

#### Nucleotide compositional skewness

Moreover, in order to capture eventual differences of skewness in the basis composition of the sequences, we computed the following skews:

(13)

Note that these measures depend on the reading frame direction, in particular their signs change with the direction. As we don't know whether right frame is direct or inverse, we could only compare *AT*_*skew *_and *CG*_*skew *_in absolute value.

#### Positional composition bias

It's known that for coding sequences in the GenBank, there is a preference for purine in the first codon position (32% G and 28% A) and for weakly bonded pair in the second position (31%A and 28% T) [11]. So we computed for each sequence the sum of densities of A-G in the first codon position and A-T in the second codon position on each reading frame:

(14)

where *n *is the number of codons in each aligned sequence, *x*_*i*1 _and *x*_*i*2 _are the bases in the 1_*st *_and 2_*nd *_position of the *i*_*th *_codon, respectively, and *I *is the indicator function, that is .

Then we selected the maximum of  among the six possible reading frames and its relative dispersion around the maximum:

(15)

(16)

#### Discrete Fourier Transform

It's known that there's a three bases periodicity in the coding DNA signal and the power spectrum at frequency of 1/3 is a measure of this periodicity [13].

In detail, each DNA sequence is converted in 4 digital signals, one for each nucleotide *α*:



where *N *is the sequence length and *n*_*j *_is the *j*_*th *_base in the sequence and 

The estimator of the power spectrum for the two signals (*α*, *β*) is defined as:

(17)

the * is the complex coniugate.

The frequency is  and *k *= 0,..., *N *- 1. To average power spectra *S*_*αβ *_(*k*), we followed the approach in [14] and defined the power spectrum of each sequence as

(18)

Finally, we computed FFT=*S*() to reveal the differences in the three-bases periodicity between the two classes.

### Predictive study

We adopted Fisher's linear classifiers [26] trained by using each feature singularly and sets of them for classifying CSEs. As measure of classifier performance we used the error rate, i. e. the fraction of both coding and non coding CSEs incorrectly predicted, or, equally, the accuracy, i. e. the fraction of both coding and non coding CSEs correctly predicted. The prediction error rate was measured by a holdout cross-validation procedure [33]. The data set was randomly split 1000 times into a training set and in a assessment set and the prediction error *E *was estimated by averaging on the 1000 errors *E*_*i *_when each sequence in the assessment set was predicted from the training set:

(19)

where *s *is the number of random splittings in training and assessment data sets.

The statistical significance of the estimated error rate *E *was assessed by using a non parametric permutation test [25]. This test aims to answer the following question: what is the probability to obtain, under the null hypothesis *H*_0 _of independent input variables and class labels, an error rate *E' *less than or equal to the really observed error rate *E*? To this end, we shuffled 1000 times the labels of the sequences and computed the P-value as the percentage of random classifiers with error rate  equal or smaller than the error *E *of the classifier trained on the correctly labelled data:

(20)

where r is the number of random label permutations. The P-value is our estimate of the probability of obtaining an error rate equal or smaller than the error *E *under the null hypothesis *H*_0_, i. e. the probability of rejecting *H*_0 _when *H*_0 _is true. Whenever the P-value was less than 0.05, we maintained that the error rate was statistically significant.

The knowledge of the empirical distribution of the error rate *E*, estimated through the cross validation procedure, allowed to evaluate an estimate of the power *π *of the test with level *α*. In fact, indicated with  the *α*-quantile of the empirical distribution *E' *of the error rate under the null hypothesis, then:

(21)

The larger is the percentage of error rates *E*_*i*_ obtained in cross-validation that are less than , the more effective is the classifier.

## Results and discussion

We characterized the distributions of feature values in coding and non-coding alignments and assessed the statistical significance of their differences using the Wilcoxon-Mann-Whitney (WMW) non-parametric test [22]. The results are summarized in Table 1. For each species, the first two columns show the mean values of each variable in the two classes and the last one shows the P-values of WMW test. The features are ranked for increasing P-values. We used the Bonferroni correction to control the probability of obtaining any false positive feature under the hypothesis that each feature is equally distributed in the two classes [34]. So we selected a cutoff value dividing 0.05 by the number of features. It results that all P-values are less than the cutoff value, i. e. the distributions of all features are significantly different in the two classes except for the *CG*_*skew *_and the %*Stop*_*std *_for M. musculus. The WMW test applied to our data set confirms some findings present in the literature. For example CG-content, frequency of A/G at first codon positions and frequency of A/T at the second codon position () are all higher for the coding sequences [10,11]. This is likely due to the higher GC content of coding exons with respect to the background sequences as well as to their compositional periodicity related to the triplet codon structure [35].

**Table 1:** The Wilcoxon-Mann-Whitney test P-values

	(a) **H. sapiens**				(b) **M. musculus**				(c) **R. norvegicus**		
**Features**	**Coding**	**Non Coding**	**P-value**	**Features**	**Coding**	**Non Coding**	**P-values**	**Features**	**Coding**	**Non Coding**	**P-values**
CG-Content	0.514	0.407	0	CG-Content	0.517	0.419	0	CG-Content	0.517	0.420	0
FFT	0.017	0.007	0	FFT	0.016	0.007	0	FFT	0.016	0.007	0
%*Stop*_*m*_	0.086	2.846	0	%*Stop*_*m*_	0.081	2.652	0	%*Stop*_*m*_	0.166	2.623	0
	0.263	0.150	0		0.264	0.153	0		0.265	0.154	0
RFC H-M	1.000	0.915	0	RFC H-M	1.000	0.915	0	RFC H-R	0.999	0.915	0
*BL*80_*M *_H-M	7.393	5.763	0	*BL*80_*M *_H-M	7.393	5.763	0	*BL*80_*M *_H-R	7.405	5.699	0
*BL*80_*std *_H-M	0.132	0.061	0	*BL*80_*std *_H-M	0.132	0.061	0	*BL*80_*std *_H-R	0.133	0.062	0
*SRR*_*M *_H-M	0.938	0.758	0	*SRR*_*M *_H-M	0.938	0.758	0	*SRR*_*M *_H-R	0.939	0.760	0
*SRR*_*std *_H-M	0.672	0.358	0	*SRR*_*std *_H-M	0.672	0.358	0	*SRR*_*std *_H-R	0.672	0.354	0
RFC H-R	0.999	0.915	0	*BL*80_*M *_M-R	7.513	6.592	0	*BL*80_*M *_M-R	7.513	6.592	0
*BL*80_*M *_H-R	7.405	5.699	0	*BL*80_*std *_M-R	0.073	0.048	0	*BL*80_*std *_M-R	0.073	0.048	0
*BL*80_*std *_H-R	0.133	0.062	0	*SRR*_*M *_M-R	0.956	0.823	0	*SRR*_*M *_M-R	0.956	0.823	0
*SRR*_*M *_H-R	0.940	0.760	0	*SRR*_*std *_M-R	0.747	0.526	0	*SRR*_*std *_M-R	0.747	0.526	0
*SRR*_*std *_H-R	0.672	0.354	0		1.178	1.094	10^-275^		1.169	1.092	10^-220^
	1.179	1.108	10^-198^	RFC M-R	0.990	0.940	10^-205^	RFC M-R	0.990	0.940	10^-205^
*AT*_*skew*_	0.155	0.129	10^-46^	NPI M-R	92.233	89.585	10^-51^	NPI M-R	92.233	89.585	10^-51^
NPI H-M	84.213	85.102	10^-14^	*AT*_*skew*_	0.157	0.132	10^-39^	*AT*_*skew*_	0.157	0.132	10^-40^
*CG*_*skew*_	0.134	0.148	10^-7^	NPI H-M	84.213	85.102	10^-15^	NPI H-R	84.248	84.448	0.0002
%*Stop*_*std*_	2.264	2.257	10^-6^	*CG*_*skew*_	0.136	0.145	0.005	%*Stop*_*std*_	2.126	2.163	0.0012
NPI H-R	84.248	84.450	0.0002	%*Stop*_*std*_	2.136	2.188	0.007	*CG*_*skew*_	0.135	0.146	0.0017

For each species, the first two columns show the mean values of each variable in the two classes and the last one shows the P-values of Wilcoxon-Mann-Whitney test. The suffices H-M, H-R and H-R indicate the species in comparison. The features are ranked for increasing P-values. Note that all P-values are less than 0.0025 except for the CG-skewness and the stop codon spread %*Stop*_*std *_for M. musculus.

Unsurprisingly, these phenomena are also much more "frame specific" for the coding sequences: in fact the spread  around the maximum  value on the different frames is greater for the coding sequences. Moreover, we observed a greater AT-skew and a smaller CG-skew in the coding regions.

The *SRR*_*M *_value is significantly higher in coding regions confirming that substitutions in the correct reading frame of homologous coding regions are more strongly biased towards synonymous changes than in any candidate reading frame in non coding alignments [17]. Furthermore, we found that nonsynonymous substitutions cause more conservative amino acid changes in the coding alignments (*BL*80_*M *_is greater in the coding pairwise alignments) [18].

The table shows that the ranking of P-values is identical for rat, human and mouse. For all three species the CG-content, FFT, %*Stop*_*m*_,  textit (intrinsic features) are the most discriminant features.

Moreover, we found that the comparative features are more discriminatory for human-mouse and human-rat alignments than for rat-mouse alignments suggesting that these features are more discriminatory at greater evolutionary distances [36].

The distributions of almost all measures being significantly different in the two classes, we might hope to attain accurate classifications by training classifiers on each feature and constructing data driven models of linear functions to discriminate coding and non coding sequences and then examine the performances of our classifiers in terms of prediction error rate on sequences that were not used to construct the model (For details see the section "Methods: Predictive study"). Nevertheless, we observed that several parameters could influence the prediction accuracy of classifiers of mRNA sequences as coding or not coding: one important parameter is the length distribution of the sequences in the training set and in the validation set.

### Sequence length dependence of classifier performances

**Figure 2:**
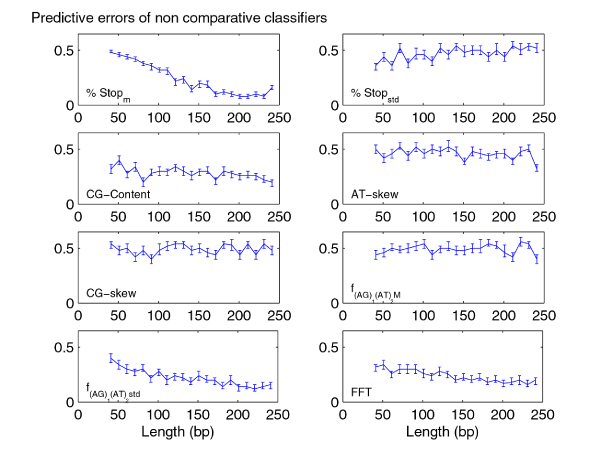
**Learning curves for the intrinsic features**. The plots refer to the error rates as function of sequence length (in bp) for H. sapiens and for each intrinsic feature.

**Figure 3:**
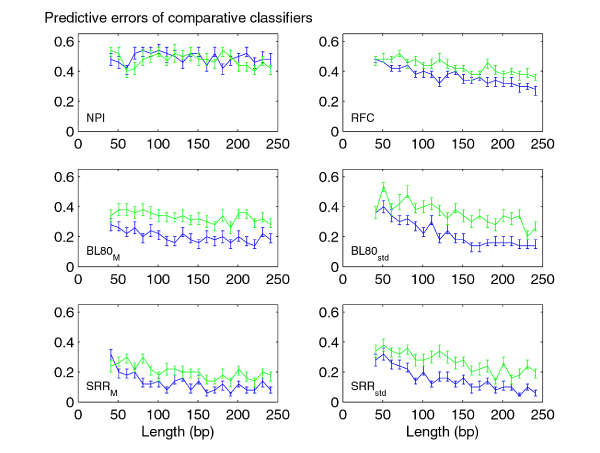
**Learning curves for the comparative features**. The plots refer to the error rates as function of sequence length (in bp) for comparative features based on the H. sapiens versus M. musculus and on the M. musculus versus R. norvegicus comparisons, respectively by blue and green lines.

We analyzed the performances of classifiers trained on rat-human-mouse alignments as a function of sequence lengths. The study of this trend gives indications about the minimum sequence length required to obtain a significantly small error rate. This analysis is especially useful for methods to identify coding and non-coding CSEs which use classifiers built on sliding windows of fixed length. Accordingly, we studied how the performances of classifiers change by varying the sequence lengths for fixed data set sizes: in particular, we controlled the predictive error rates monitoring their P-values and the powers of classifiers (See "Methods: Predictive study"). To this end, we built 21 different balanced data sets, that is with coding and non coding alignments in equal number (75 coding and 75 non coding sequences), and with equally long sequences with an ascending average sequence lengths. The sequence lengths of the 21 data sets were respectively in the following ranges: [41, 50], [51,60], . . . , [241,250] bp. For each classifier and for each species, we constructed learning curves in which the empirical error rate (median, 25% and 75% quantiles) is plotted as a function of sequence length. As the trends of learning curves related to the non comparative features were very similar for all three species, we reported in Figure 2 only the results for human sequences. Concerning the comparative features, we observed that the learning curves for human versus rat comparison are very similar to those for human versus mouse. Accordingly, the Figure 3 shows only the human/mouse and mouse/rat curves. Several features show significantly different distributions for coding and non-coding sequences/alignments but are not able to accurately discriminate between the two classes of sequences (P-values > 0.05). These features include *CG*_*skew*_, *AT*_*skew*_, , %*Stop*_*std *_and NPI.

For the intrinsic features with statistically significant error rates (%*Stop*_*m*_, CG-Content, , FFT) we observed decreasing error rates with ascending sequence lengths (see Figure 2). The same behavior was observed for comparative features with statistically significant error rates, i. e. for the Blosum scores *BL*80_*M *_and *BL*80_*std*_, the substitution rate ratio scores *SRR*_*M *_and *SRR*_*std *_and the reading frame conservation RFC. Moreover, our results suggest that these comparative features better discriminate for more genetically distant species. Error rates related to the human/mouse and human/rat comparisons (blue line) are smaller than those obtained in the mouse/rat comparison (green line).

**Figure 4:**
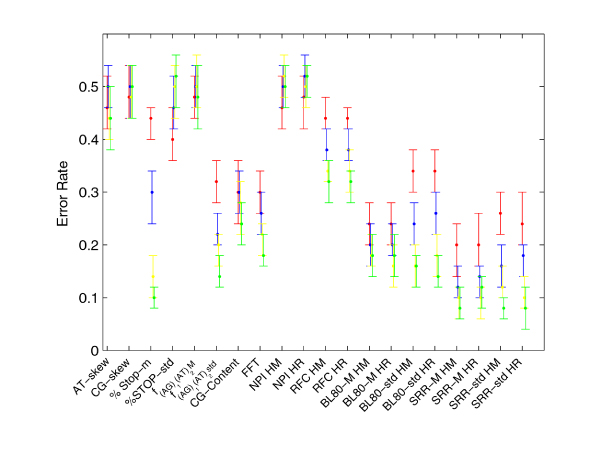
**The summary plot**. The error bars in the figure represent the median, 25% and 75% quantiles error rates for H. sapiens sequences and for their pairwise alignments: the red, blue, yellow and green bars refer to the 4 classes of ascending sequence lengths in the legend.

In order to compare the performance of all classifiers according to the sequence lengths, we summarized the results for human sequences and their alignments in a unique plot shown in Figure 4. To build this summary plot, we grouped the empirical estimated error rates in four classes by averaging error rates of the classifiers related to the increasing sequence lengths: [41,90[, [91,140[, [141,190[, [191,250] bp. Our analysis suggests that the percentage of stop codons %*Stop*_*m*_, the rate ratio *SRR*_*M *_and its spread *SRR*_*std *_are the most discriminatory features for the species considered. Nevertheless, %*Stop*_*m *_is strongly influenced by the sequence lengths, while *SRR*_*std *_and *SRR*_*m *_exhibit less than 30% error rates even for sequences and alignments with lengths in the [41, 90[base range. Finally, we point out the behavior of the classifier trained by using Blosum scores *BL*80_*M *_which provides small error rates independently of the sequence lengths. Lower performance is expected for all methods when confronted with short sequences due to stochastic factors, the poor performance of the %*Stop*_*m *_metric with short sequences is unsurprising given that low numbers of stop codons are expected even for non-coding sequences when the length is short. In order to obtain higher accuracies, for each sequence length we trained a classifier with the comparative features and a classifier with the intrinsic ones.

**Figure 5:**
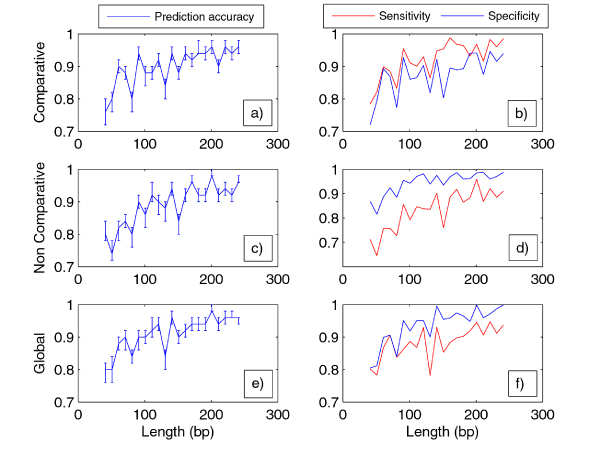
**Sensitivity and specificity**. The plots refer to the sequences of the H. sapiens and their alignments with rat and mouse genomes: in particular on the right the are the three plots of prediction accuracy of the combination of the only comparative a), of the only intrinsic c) and of all metrics e) as function of sequence lengths, on the left the respective plots b), d), f) for the sensitivity and the specificity.

The features depending on the reading frame were evaluated by using the frame suggested by the most accurate univariate classifier, i. e. the classifier based on *SRR*_*M*_. The empirical distributions (median and interquartile range) of prediction accuracies of comparative and intrinsic classifiers are depicted respectively in Figure 5a) and 5c). Both learning curves are ascending for increasing sequence length: the accuracies vary in [70%, 97%] and are statistically significant for each sequence length (*P-value *< 0.005 and *π *> 0.88). Although these learning curves exhibit a similar qualitative behavior, they result statistically different. In fact, Kolmogorov-Smirnov tests show that the error rates of comparative classifiers are significantly smaller than ones of non comparative classifiers in 68% of the length classes for the human, in 65% for the mouse and in 94% for the rat.

Moreover, we investigated if adding the intrinsic features to the comparative ones could improve the performance of the classifier in a statistically significant way. To this end we trained a classifier by using the above features simultaneously and assessed its error rate as a function of the sequence length. For each length class, the empirical distribution (median and interquartile range) of prediction accuracy of this *global *classifier is depicted in Figure 5e). By the permutation test, the significance and the predictive power of the error rate were assessed: all 21 error rates are significant with *P-values *< 0.05 and *π *> 0.992. The accuracy increases proportionally to the sequence length up to 97% (*P-value *= 0, *π *= 1). Kolmogorov-Smirnov tests, applied for comparing comparative versus global classifiers, show that the error distribution of the global classifier is significantly smaller than the one of the comparative classifier, for all three species in analysis and independently of the sequence length. In other terms, the information related to the intrinsic features is not redundant in the ensemble of all features in order to classify coding from non coding CSEs.

We completed our analysis comparing the global, comparative and intrinsic classifiers in terms of sensitivity (proportion of coding sequences/alignments classified as coding) and specificity (the fraction of non-coding sequences correctly predicted as non-coding) (see Figure 5b), 5d) and 5f)). We found that the combination of comparative features is more powerful in the classification of protein coding sequences while the inverse is true for the intrinsic features independently on sequence length. In the global classifier prevails the non comparative behavior: it better predicts the non coding than the coding CSEs regardless of sequence length. The fact that comparative features tend to be more accurate in the classification of coding sequences is likely related to the fact that such features look deviations from strictly neutral evolution expected as a consequence of the characteristics of the genetic code. Conversely, novel intrinsic features might be expected to aid in the correct classification of non-coding sequences.

We still do not know the complete inventory of human and other eukaryotic genomes although several efforts in this direction have been carried out so far. Phylogenetic footprinting is a powerful tool for such purpose as evolutionary conservation is a significant hallmark of protein coding potential. Indeed, coding portions of genes generally are under strong selective pressure that preserve their primary sequence [37]. However, also some non coding regions are highly conserved or ultraconserved as they may be involved in transcriptional and post-transcriptional regulatory activity [38]. Thus the problem is to reliably discriminate between coding and non-coding conserved sequences, as the first, when falling outside current annotations, may represent novel exons of alternative splicing variants or of unknown protein coding genes, while the latter are likely involved in some regulatory activity. We previously developed an heuristic method to measure the protein coding potential that resulted quite well performing [18,39]. However, we did not evaluate the specific contributions of different features on the discriminatory efficiency. The results presented here fill this gap as they measure the predictive potential of different features, both those based on intrinsic features simply based on primary sequences (e.g. base composition or periodicity) and those deriving from comparative measures (e.g. Ks or Ka). We also evaluated the effect of CSE length on the prediction accuracy. Results presented reveal some general statistic properties of coding and non coding sequences that may be of general interest also for other studies aimed at their classification adopting different methodologies.

## Conclusion

In this paper we have provided a systematic and statistically well founded assessment of various comparative and non-comparative features for distinguishing coding from regulatory motifs in conserved sequences tags among human, rat and mouse species. In our study we evaluated the relevance of single as well as groups of features in distinguishing coding from non coding CSEs by using association and prediction studies. The distributions of the analyzed features were statistically different in the two classes, confirming well known results and suggesting novel differences between coding and no coding CSEs which should be confirmed on different data sets. Moreover, we have provided an experimental evidence concerning the relevance of intrinsic features in predicting cross-species alignments. In fact, the prediction accuracy of classifiers trained by using comparative features increased significantly by adding intrinsic features to the set of input variables. We observed distinct and consistent patterns for individual and combined use of comparative and intrinsic classifiers, both with respect to different length classes of sequences/alignments and with respect to error rates in the classification of coding and non-coding exemplars.

A problem, worthy of future study, is derived from the fact that most, if not all, published comparative methods have been trained and evaluated with entirely coding or entirely non-coding alignments. While this renders generation of training sets more tractable, it does not reflect the real situation encountered during the annotation of draft genome or other sequences where alignments may be generated through similarity searches, but no a-priori information regarding delineation of coding regions is available. To this end, our data showing that carefully chosen features can show high sensitivity and specificity even for short alignments suggest that sliding window approaches may be capable of addressing this issue.

Finally, it is clear that while, particularly for the comparative features, different measures are far from independent (all such features measure deviations from random substitution patterns expected as a consequence of the genetic code), different features function differentially at different evolutionary distances. In general therefore, it should be considered desirable to use multiple species comparisons spanning different levels of divergence – both in order to maximize the proportion of the reference genome which is aligned, and to maximize the discriminatory power of the tools at hand. Such approaches are the subject of ongoing work.

## Competing interests

The authors declare that they have no competing interests.

## Authors' contributions

NA and GP conceived the study. TMC, FM, AD'A and RM designed the algorithms and conduced the experiments and, together with DSH, GP and NA, they evaluated and compared the experimental results. All the authors contributed to the drafting of the article.

## References

[B1] Stark A (2007). Discovery of functional elements in 12 Drosophila genomes using evolutionary signatures. Nature.

[B2] Consortium MGS (2002). Initial sequencing and comparative analysis of the mouse genome. Nature.

[B3] Consortium RGSP (2004). Genome sequence of the Brown Norway rat yields insights into mammalian evolution. Nature.

[B4] Yang S, Smit AF, Schwartz S, Chiaromonte F, Roskin KM, Haussler D, Miller W, Hardison RC (2004). Patterns of insertions and their covariation with substitutions in the rat, mouse, and human genomes. Genome Research.

[B5] Jensen-Seaman MI, Furey TS, Payseur BA, Lu Y, Roskin KM, Chen CF, Thomas MA, Haussler D, Jacob HJ (2004). Comparative Recombination rates in the rat, mouse and human genomes. Genome Research.

[B6] Kellis M, Patterson N, Birren B, Berger B, Lander ES (2004). Methods in comparative genomics: genome correspondence, gene identification and regulatory motif discovery. J Comput Biol.

[B7] Noguchi H, Yada T, Sakaki Y (2002). A novel index which precisely derives protein coding regions from cross-species genome alignments. Genome Informatics.

[B8] Rivas E, Eddy S (2001). Noncoding RNA gene detection using comparative sequence analysis. BMC Bioinformatics.

[B9] Mignone F, Grillo G, Liuni S, Pesole G (2003). Computational identification of protein coding potential of conserved sequence tags through cross-species evolutionary analysis. Nucleic Acids Res.

[B10] Bibb ML, Findlay PR, Johnson MW (1984). The relationship between base composition and codon usage in bacterial genes and its use for the simple and reliable identification of protein-coding sequences. GENE.

[B11] Buldyrev SV (2005). Power Law Correlations in DNA Sequences. Eurekah Bioscience Collection.

[B12] Fickett JW (1982). Recognition of protein coding regions in DNA sequences. Nucleic Acids Research.

[B13] Anastassiou D (2001). Genomic Signal Processing. IEEE Signal Processing Magazine.

[B14] Voss R (1992). Evolution of long-range fractal correlations and 1/f noise in DNA base sequences. Phys Rev Lett.

[B15] Fickett JW, Tung CS (1992). Assessment of protein coding measures. Nucleic Acids Research.

[B16] Gao F, Zhang CT (2004). Comparison of various algorithms for recognizing short coding sequences of human genes. Bioinformatics.

[B17] Nekrutenko A, Makova K, Li WH (2002). The KA/KS ratio test for assessing the protein-coding capacity of genomic regions: An emprirical and simulation study. Genome Research.

[B18] Castrignanò T, Canali A, Grillo G, Liuni S, Mignone F, Pesole G (2004). CSTminer: a web tool for the identification of coding and noncoding conserved sequence tags through cross-species genome comparison. Nucleic Acids Research.

[B19] Badger JH, Olsen GJ (1999). CRITICA: Coding region identification tool invoking comparative analysis. Mol Biol Evol.

[B20] Liu J, Gough J, Rost B (2006). Distinguishing protein-coding from non-coding RNAs through support vector machines. PLoS Genet.

[B21] Vapnik V (1995). The Nature of Statistical Learning Theory.

[B22] Hollander M, Wolfe DA (1999). Nonparametric statistical methods.

[B23] Mukherjee S, Tamayo P, Rogers S, Rifkin R, Engle A, Campbell C, Golub TR, Mesirov JP (2003). Estimating dataset size requirements for classifying DNA microarray data. J Comput Biol.

[B24] Michiels S, Koscielny S, Hill C (2005). Predictor of cancer outcome with microarrays: a multiple random validation strategy. Lancet.

[B25] Good P (1994). Permutation tests: a practical guide to resampling methods for testing hypotheses.

[B26] Anderson TW (1958). An introduction to multivariate statistical analysis.

[B27] Kent W (2002). BLAT-the BLAST-like alignment tool. Genome Res.

[B28] Altschul S, Madden T, Schaffer A, Zhang J, Zhang Z, Miller W, Lipman D (1997). Gapped BLAST and PSI-BLAST: a new generation of protein database search programs. Nucleic Acids Research.

[B29] Nei M, Gojobory T (1986). Simple Methods for Estimating the Numbers of Synonymous and Nonsynonymous Nucleotide Substitutions. Mol Biol Evol.

[B30] Nei M, S K (2000). Synonymous and nonsynonymous nucleotide substitutions. Molecular Evolution and Phylogenetics.

[B31] Jukes TH, Cantor CR, Munro HN (1969). Evolution of protein molecules. Mammalian protein metabolism III.

[B32] Henikoff S, Henikoff JG (1992). Amino acid substitution matrices from protein blocks. Proc Natl Acad Sci USA.

[B33] Davison AC, Hinkley DV (1997). Bootstrap methods and Their Application.

[B34] Ewens WJ, Grant GR (2004). Statistical Methods in Bioinformatics.

[B35] Aissani B (1991). The compositional properties of human genes. J Mol Evol.

[B36] Lin MF, Deoras AN, Rasmussen MD, Kellis M (2008). Performance and Scalability of Discriminative Metrics for Comparative Gene Identification in 12 Drosophila Genomes. Plos computational biology.

[B37] Ganley A, Kobayashi T (2007). Phylogenetic footprinting to find functional DNA elements. Methods Mol Biol.

[B38] Siepel A (2005). Evolutionarily conserved elements in vertebrate, insect, worm, and yeast genomes. Genome Res.

[B39] Castrignanò T, Meo PDD, Grillo G, Liuni S, Mignone F, Talamo I, Pesole G (2006). GenoMiner: a tool for genome-wide search of coding and non-coding conserved sequence tags. Bioinformatics.

